# Practicing Level and Determinants of Safe Cord Care and Skin-To-Skin Contact Among Post-partum Women in Public Hospitals of Eastern Ethiopia

**DOI:** 10.3389/fped.2022.883620

**Published:** 2022-06-02

**Authors:** Addis Eyeberu, Tamirat Getachew, Adera Debella, Abdi Birhanu, Addisu Alemu, Yadeta Dessie

**Affiliations:** ^1^School of Nursing and Midwifery, College of Health and Medical Sciences, Haramaya University, Harar, Ethiopia; ^2^School of Medicine, College of Health and Medical Sciences, Haramaya University, Harar, Ethiopia; ^3^School of Public Health, College of Health and Medical Sciences, Haramaya University, Harar, Ethiopia

**Keywords:** newborn, cord care, skin-to-skin contact (SSC), Ethiopia, neonate

## Abstract

**Background:**

Even though practicing levels of safe cord care and skin-to-skin contact among post-partum women are critical to reducing neonatal deaths, limited data revealed the low practice. Thus, the purpose of this study was to determine the level of practice and determinants of safe cord care and skin-to-skin contact among post-partum women in public hospitals of Eastern Ethiopia.

**Methods:**

A facility-based cross-sectional study was conducted at the public hospitals of Harari reginal state, eastern Ethiopia. A random sample of 820 post-partum women was included in the study. A pre-tested and structured questionnaire was used to collect data through a face-to-face interview. STATA version 14 was used for data analysis. Bivariable and multivariable logistic regression analyses were employed to determine the association between independent and outcome variables.

**Results:**

The practicing level of safe cord care was 71.7% (95% Confidence Interval (CI): 64.5, 81.7). While the practicing level of Skin-To-Skin contact was 53.2% (95% CI: 43.6, 58.8). Being in age of 20–29 [adjusted odds ratio (AOR) = 2.93, 95% CI: 1.24, 6.96], attending tertiary education [AOR = 1.83, 95% CI (1.08, 3.13)], and having good knowledge about safe cord care [AOR = 11.3, 95% CI: (7.49, 17.18)] were determinants of safe cord care practice. While mothers aged 20–29, 30–39, and above 40 [(AOR = 11.17, 95% CI: 4.71, 26.5; AOR = 4.1, 95% CI: 1.77, 9.55, and AOR = 14.3, 95% CI: 7.2, 28.6), respectively], Being married [AOR = 3.70, 95% CI (1.58, 8.70)], being a merchant and self-employed ([AOR = 0.55, 95% CI: 0.34,0.87] and [AOR = 0.49, 95% CI: 0.27, 0.86], respectively), having good knowledge about SSC [AOR = 2.11, 95% CI: (1.53, 2.92)], giving birth at gestational age of 37–42 weeks [AOR = 1.82, 95% CI (1.31, 2.5)], and multigravidity (AOR = 2.83, 95% CI (1.90,4.21) were significantly associated with skin to skin contact.

**Conclusions:**

The practicing level of safe cord care and skin-to-skin contact was high. In this study, the age of mothers, educational status, and knowledge of post-partum women on safe cord care were determinants of a safe cord care practice. While the age of mothers, marital status, occupational status, knowledge of mother, and gestational age at birth were significantly associated with skin-to-skin contact practice. Safe cord care should be strengthened and intensified to reduce neonatal mortality due to avoidable umbilical cord infections. Furthermore, skin-to-skin contact practice should be strengthened to enhance the survival of at-risk neonates.

## Introduction

Comprehensive and standard newborn care practices are critical interventions for reducing approximately two-thirds of neonatal deaths (1–3). Safe cord care and skin-to-skin contact between the mother and her baby immediately after birth are included as part of these interventions, and they benefit both maternal and infant health (1, 4).

Skin-to-skin contact, both psychologically and physiologically, is thought to be a “sensitive period” for determining future physiology and behavior. It is a non-pharmacological treatment for discomforts that involves the fulfillment of basic biological demands and is capable of inducing neurobehavioral changes (5–7). Standardization of these practices is crucial for the physiological and psychological wellbeing of mothers and infants (8). Proper newborn care, such as safe cord care and skin-to-skin contact, is especially important for the at-risk newborn in resource-limited settings (9). Among the benefits are the stimulation of breastfeeding patterns, the stabilization of the newborns' body temperature, and the improvement of bonding (10, 11), and decreasing the maternal depressive symptoms and physiological stress during the post-partum time are vital ones (12, 13).

Even though skin-to-skin contact between neonates and their mothers has numerous benefits for improving the baby's survival, it is being poorly practiced, particularly in resource-limited settings (14). According to studies, Ethiopia has a low SSC practice that ranges from 28.1 to 35.3% in different parts of the country (15, 16). Several studies reported that factors such as maternal education, number of ANC visits, the residence of the family, newborn birth weight, time of breastfeeding initiation after delivery, optimum breastfeeding content-wise, maternal knowledge status toward skin-to-skin contacts, maternal and infant benefits, preterm, Apgar score, route of delivery, the health status of the newborn, place of delivery, and late initiation of ANC follow-up affect the level of skin-to-skin practices (14–18).

A study found that only 46.9% of neonates had a safe cord practice (1). The safe cord care practice could be determined by factors such as maternal age, maternal educational level, sex of the delivered baby, place of delivery, awareness about hygienic safe cord care, source of knowledge about safe cord care, maternal occupation, and parity (2, 19, 20).

Despite the fact that safe cord care and skin-to-skin contact are important in reducing neonatal deaths, limited data revealed a lack of practice. Furthermore, the determinants of low implementation of these interventions in Ethiopia are not well-understood. Evidence also suggests that quantitative data on skin-to-skin contact (SSC) is extremely scarce in Sub-Saharan African countries and should be investigated. Thus, the purpose of this study was to determine the level of practice and determinants of safe cord care and skin-to-skin contact among post-partum women in public health facilities in Eastern Ethiopia.

## Materials and Methods

### Study Design, Study Setting, and Period

A facility-based cross-sectional study was conducted from May 20 to June 10, 2021, at public hospitals in Harari reginal state, eastern Ethiopia [Hiwot Fana Specialized University Hospital (HFSUH) and Jugol hospital]. The region is found 526 km far from Addis Ababa, the capital city of Ethiopia. The region is circled by Oromia regional state. The region is divided into nine woredas, three of which are rural and six of which are urban. The urban districts are divided into 19 kebeles (the country's lowest administrative unit), while the rural districts are divided into 17 peasant associations (which is equivalent to kebeles in urban cases). The region's total population (2021 projection based on the 2007 Census, CSA) is 270,000, with 136,000 males and 134,000 females (1). There are 59,487 total households (21). There are two public hospitals, two private hospitals, eight public health centers, and 20 health posts in the region. HFSUH serves as a referral hospital in eastern Ethiopia. The obstetrics ward encompasses labor and delivery, maternity, and post-natal units and the ward has a total of 54 beds. Jugol hospital was established in 1902 G.C. and it serves more than 500,000 populations.

### Study Population and Eligibility Criteria

Post-partum women who came for immunization services at public hospitals in eastern Ethiopia during the data collection period were the study populations. All selected post-partum mothers-child pairs were included while mothers who were unable to respond to the survey and were seriously ill were excluded.

### Sample Size Determinations and Sampling Techniques

The sample size was calculated using the single population proportion formula and double population proportion formulas. However, the largest sample size was obtained using the single population formula. The sample size was calculated as follows.


$$\begin{array}{l} \text{n}=\left(Z\alpha /2\right)2p\;\left(1-p\right)/d2 \end{array}$$

*n* = the minimum sample size required, p = estimated proportion of Safe cord care practice Zα/2 = the value of standard score at 95% confidence interval (1.96); with the following assumptions: Confidence level at 95% = 1.96; Margin of error (d) = 0.05 and non-response rate = 10% is considered to get appropriate sample size with available resource and prevalence of safe cord care 46.9% from Dessie referral hospital, northeast Ethiopia (1). The sample size was calculated as (Zα/**2**) 2^*^ P(1-q)/d2 = (1.96)2 ^*^ 0.469^*^ 0.531/ (0.035)2 = 781 by adding non-response rate (10%), final sample size was 859.

Hiwot Fana Specialized Hospital and Jugol hospitals as they are the only public hospitals in the region. The determined sample was allocated proportionally to each Hospital based on the childbirth flow throughout the year before the data collection. Accordingly, 612 from HFSUH and 247 from Jugol Hospital mother-child pairs were included in the study. The systemic sampling method was implemented in each Hospital separately based on monthly childbirth flow and in an interval of three was taken by calculating the sampling fraction. The first case was selected randomly by the lottery method.

### Data Collection Methods

A structured interview administrated questionnaire adopted from previous similar studies was used to collect data (22). The questionnaire includes socio-demographic characteristics of the study participants, knowledge of mothers on SSC and CC, and the practice of SSC and CC. The tool was prepared in English language and translated to the local language in the study area and again translated back to the English language to check the consistency. The data were collected by well-trained six professional Nurses and Midwives and supervised by two MSC-holding Midwives. The quality of the data was guaranteed by pretesting using the 5% of the respondents who were not eligible for the study before actual data collection. The completeness of the data was checked daily.

### Measurements

#### Cord Care

Keeping the cord clean and dry without application of any foreign substances until the umbilical stump falls off (until 7 days of life) (23). In this study, the mother's cord care was classified as clean when it was kept dry and free of any foreign substances; other forms of treatment to the umbilical cord stump were classified as unclean, including the use of butter, herbs, salt, oil, and Vaseline (24, 25). In this study, safe cord care practice was measured dichotomously as “clean cord care” coded as 1 and “unclean cord care” coded as 0.

#### Skin-To-Skin Contact

Placing an unclothed or diaper only newborn baby on the mother's bare chest or abdomen soon after birth and keeping them together continuously for at least 1 h regardless of the mode of delivery, place of delivery, and feeding methods (16, 26). In this study, skin-to-skin care practice was measured dichotomously as “Yes” coded as 1 and “No” coded as 0 (14).

Mothers' knowledge of skin-to-skin contact and safe cord care was assessed using eight and seven items, respectively. During analysis, the correct answer was coded as “1,” while the incorrect or I do not know the answer was coded as “0.” The sum score was then computed and categorized. Mothers who answered correctly above the mean on knowledge-related questions were categorized as having good knowledge, while mothers who answered incorrectly below the mean on knowledge-related questions were categorized as having poor knowledge (16, 22, 27).

### Statistical Analysis

The data was coded, cleaned, edited, and entered into Epi data statistical software version 3.1 and then exported to STATA version 14 for analysis. Descriptive statistical analysis such as simple frequency was used to describe the characteristics of study participants such as socio-demographic, and knowledge of mothers. Then the information was presented using frequencies and tables.

Bivariate analysis and multivariate analysis were done to see the association between each independent variable and outcome variables by using binary logistic regression. The assumptions of binary logistic regression were cheeked. The goodness of fit was checked by Hosmer-Lemeshow statistic and omnibus tests. All variables with *P* < 0.25 in the Bivariate analysis were included in the final model of multivariate analysis to control all possible confounders. The multi co-linearity test was carried out to see the correlation between independent variables by using the standard error and collinearity statistics (variance inflation factors >10 and standard error >2 were considered suggestive of the existence of multi co-linearity). The direction and strength of statistical association were measured by odds ratio with 95% CI. Adjusted odds ratio along with 95% CI was estimated to identify the association between independent variables and SSC and CC by using multivariable analysis in binary logistic regression. In this study, *P*-Value <0.05 was considered to declare a result statistically significant.

## Results

### Socio-Demographic Characteristics

Of a total of 859 sampled post-partum mothers, 820 were involved in the study providing a 95.5% response rate. Five hundred twenty-six (64.1%) of post-partum mothers were Muslim followers while 264 (32.2%) of them were Orthodox Christian followers. The majority of the post-partum mothers were Oromo 90.2%, while 44 (5.37%) of them were Amhara. Among post-partum mothers, 91, 53.4, and 52.4% of them were married, in the age group of 20–29, and housewives, respectively (Table 1).

**Table 1:** Sociodemographic characteristics of post-partum women at public hospitals of eastern Ethiopia, 2021.

**Variable**	**Category**	**Skin-to-skin contact care**		***P*** **-value**	**Cord care**		***P*** **-value**
		**Yes (%)**	**No (%)**		**Clean**	**Unclean**	
Age of mothers	15–19	42 (84)	8 (16)	1	42 (84)	8 (16)	1
	20–29	256 (58.4)	182 (41.6)	≤ 0.01	296 (67.6)	142 (32.4)	0.02
	30–39	130 (44.2)	164 (55.8)	≤ 0.01	222 (75.5)	72 (24.5)	0.19
	Above 40	8 (21)	30 (79)	≤ 0.01	28 (73.7)	10 (26.3)	0.24
Marital status	Single	10 (26.3)	28 (73.7)	1	32 (84.2)	6 (15.8)	1
	Divorced/widowed	16 (44.4)	20 (55.6)	0.11	34 (94.4)	2 (5.6)	0.17
	Married	410 (55)	336 (45)	≤ 0.01	522 (70)	224 (30)	0.07
Educational status	No formal education	88 (60.3)	58 (39.7)	1	46 (28.8)	104 (71.2)	1
	Primary	94 (69.1)	42 (30.9)	0.122	32 (24.2)	100 (75.8)	0.14
	Secondary	140 (55.6)	112 (44.4)	0.359	72 (28.6)	180 (71.4)	0.54
	College and above	114 (39.9)	172 (60.1)	≤ 0.01	82 (28.7)	204 (71.3)	0.54
Occupational status	Housewife	264 (61.4)	166 (38.6)	1	142 (33)	288 (77)	1
	Merchant	44 (40.7)	64 (59.3)	≤ 0.01	12 (11.1)	96 (88.9)	≤ 0.01
	Self-employee	40 (51.3)	38 (48.7)	0.095	18 (23.1)	60 (76.9)	0.08
	Governmental employee	88 (43.1)	116 (56.9)	≤ 0.01	60 (29.4)	144 (70.6)	0.36

### Health Service Utilization

During their pregnancy, 716 (87.3%) of post-partum women had ANC visits. Five Hundred thirty-four (66.6%) of those who had ANC visits had three or fewer ANC visits, while 268 (33.4%) had four or more ANC visits. More than half of the 448 women (55.9%) had their ANC follow-up at a health center, while 37.2 and 6.9% had their ANC visits at public hospitals and private health institutions, respectively. Among post-partum women, 30.4% delivered their first baby at the age of below 18. Thirty-five (4.3%) of the women deliver their baby at home. More than three fourth (79.5%) of women deliver their baby through spontaneous vaginal delivery (Table 2).

**Table 2:** Health service utilization among post-partum women at public hospitals of Eastern Ethiopia, 2021.

**Variable**	**Category**	**SSC**		***P*** **-value**	**CC**		***P*** **-value**
		**Yes**	**No**		**Clean**	**Unclean**	
ANC follow-up	Yes	387 (54)	329 (46)	0.186	200 (27.9)	516 (72.1)	0.55
	No	49 (47.1)	55 (52.9)	1	32 (30.8)	72 (69.2)	1
Gravidity	Primigravida	160 (50.3)	158 (49.7)	1	94 (29.6)	224 (69.4)	1
	Multigravida	276 (55)	226 (45)	0.192	138 (27.5)	364 (72.5)	0.15
History of baby loss	Yes	48 (54.5)	40 (45.5)	1	30 (34.1)	58 (65.9)	1
	No	388 (53)	344 (47)	0.784	202 (27.6)	530 (72.4)	0.20
Age at first birth	Below 18	196 (79)	52 (21)	1	60 (24.2)	188 (75.8)	1
	Above 18	240 (42)	332 (58)	≤ 0.01	172 (30)	400 (70)	0.09
Gestational age at birth	Below 37 weeks	120 (42.9)	160 (57.1)	1	74 (26.4)	206 (73.6)	1
	37–42 weeks	306 (59.5)	208 (40.5)	≤ 0.01	142 (27.6)	372 (72.4)	0.72
	Above 42 weeks	10 (38.5)	16 (61.5)	0.665	16 (61.5)	10 (38.5)	≤ 0.01
Place of delivery	Home	12 (34.3)	23 (65.7)	1	12 (34.3)	23 (65.7)	1
	Health institution	424 (54)	361 (46)	0.025	220 (28)	565 (72)	0.24
Mode of delivery	SVD	379 (58)	273 (42)	0.26	180 (27.6)	472 (72.4)	0.27
	Instrumental	34 (52.3)	31 (47.7)	0.31	28 (43)	37 (57)	0.40
	C/S	23 (33.8)	45 (66.2)	1	24 (35.3)	44 (64.7)	1
Immediate PP skin-to-skin care counseling	Yes	394 (52.4)	358 (47.6)	0.140	202 (26.8)	550 (73.2)	0.03
	No	42 (61.8)	26 (38.2)	1	30 (44)	38 (56)	1
Immediate PP cord care counseling	Yes	388 (53)	346 (47)	0.604	202 (27.5)	532 (72.5)	0.15
	No	48 (55.8)	38 (44.2)	1	30 (34.9)	56 (65.1)	1

*ANC, antenatal care; PP, post-partum; SVD, spontaneous vaginal delivery; C/S, caesarian section*.

### The Magnitude of Safe Cord Care and Skin-To-Skin Contact Practices

Among post-partum women, 588 (71.7%) of them practiced clean cord care after delivery. So, the practicing level of safe cord care (CC) was 71.7% (95% CI: 64.5, 81.7). Among 820 post-partum women, 436 (53.2%) of them placed their babies in skin-to-skin contact in the first 1 h after delivery. So the magnitude of Skin-To-Skin care (SSC) was 53.2% (95% CI: 43.6, 58.8).

Among those mothers who were not practiced clean cord care, 88 (10.73%) applied butter on the cord, 52 (6.34%) vaseline, 60 (7.32%) petroleum jelly, and 32 (3.9%) others (hair lotion, ointment).

### Knowledge of Mothers on Safe Cord Care and SSC

Among interviewed mothers, 774 (94.3%) mothers said that they knew how to care for the cord. Among those mothers who were asked how to handle umbilical cord, 404 (49.27%) of them handle umbilical cord with dressing, and 52 (5.61%) were handled without dressing while 364 (44.39%) of them don't know how to handle umbilical cord. Among mothers who were asked what to do if the cord bleeds, most (98.8%) of them would go to a health center while six, and four of them would apply home medications and wait until it healed by itself, respectively. Among mothers asked about the benefit of safe cord care, more than three fourth (76.3%) of them knew that it prevents infection (21.3%) quick cord separation, and (19.4%) prevent abdominal pain.

Among mothers who were asked about the advantage of skin-to-skin contact, 45.5% of them knew that it facilitates breastfeeding, 34.4% of them knew that it increases feelings of bonding and attachment, and 12.3% of them knew that it prevents hypothermia.

The mean score of knowledge of mothers on SSC was 4.3 (SD = 1.2) with a minimum and maximum score of 0 and 7, respectively. About 424 (51.7%) of the study participants had good knowledge regarding SSC. The mean score of knowledge of mothers on safe cord care was 3.8 (SD = 1.5) with a minimum and maximum score of 1 and 6, respectively. About 451 (55%) of the study participants had good knowledge regarding safe cord care.

### Determinants of Skin-To-Skin Contact Care and Safe Cord Care Practices

#### Determinants of Skin-To-Skin Contact Care

Variables such as the age of mothers, marital status, educational status, occupational status, age at first birth, gestational age at birth, and knowledge of post-partum women on SSC and CC were all significantly associated with Skin-To-Skin Contact Care (SSC) in the binary logistic regression model. The final model included variables that met the multivariable logistic regression assumption. A multivariable logistic regression, maternal age, marital status, occupational status, gravidity, gestational age at birth, and post-partum women's knowledge of SSC and CC were all significantly associated with SSC practice.

In this study, the mother's age was found to be significantly associated with SSC practice. Mothers aged 20–29, 30–39, and over 40 were 11.17, 4.1, and 14.3 times more likely to practice skin-to-skin contact care than mothers aged 19 and under [(AOR = 11.17, 95% CI: 4.71, 26.5), (AOR = 4.1, 95% CI: 1.77, 9.55; AOR = 14.3, 95% CI: 7.2, 28.6)]. The odds of SSC care practice were 3.70, 2.83, and 1.82 times higher in married, multigravida, mothers with a gestational age of 37–42 weeks than in single, primigravida, and mothers with a gestational age of lower than 37 weeks [AOR = 3.70, 95% CI (1.58, 8.70)], [AOR = 2.83, 95%CI (1.9, 4.21)], and [AOR = 1.82, 95% CI (1.31, 2.5)], respectively. In this study, mothers whose occupational status was a merchant and self-employed were 0.55 and 0.49 times less likely to practice SSC care, respectively [AOR = 0.55, 95%CI (0.34,0.87) (AOR = 0.49, 95%CI: 0.27,0.86)] than mothers whose occupational status was a housewife. Mothers with good knowledge levels were 3.19 times more likely to practice SSC care as compared to respondents with poor knowledge levels [AOR = 2.11, 95%CI: (1.53, 2.92)] (Table 3).

**Table 3:** Determinants of Skin-to-skin contact practice among post-partum women at public hospitals in eastern Ethiopia, 2021.

**Variable**	**Category**	**Skin-to-skin contact care**		**COR 95% CI**	**AOR 95% CI**	***P*** **-value**
		**Yes**	**No**			
Age of mothers	15–19	42 (84)	8 (16)	1	1	1
	20–29	256 (58.4)	182 (41.6)	3.73 (1.71, 8.14)	11.17 (4.71, 26.5)	≤ 0.01
	30–39	130 (44.2)	164 (55.8)	6.62 (3.01, 14.59)	4.1 (1.77, 9.55)	≤ 0.01
	Above 40	8 (21)	30 (79)	19.7 (6.64, 58.33)	14.3 (7.2, 28.6)	≤ 0.01
Marital status	Single	10 (26.3)	28 (73.7)	1	1	1
	Divorced/widowed	16 (44.4)	20 (55.6)	0.45 (0.168, 1.19)	1.16 (0.38, 3.56)	0.80
	Married	410 (55)	336 (45)	0.29 (0.14, 0.611)	3.7 (1.58, 8.70)	0.03
Occupational status	Housewife	264 (61.4)	166 (38.6)	1	1	1
	Merchant	44 (40.7)	64 (59.3)	2.31 (1.50, 3.56)	0.55 (0.34, 0.87)	≤ 0.01
	Self-employee	40 (51.3)	38 (48.7)	1.51 (0.93, 2.45)	0.49 (0.27, 0.86)	≤ 0.01
	Governmental employee	88 (43.1)	116 (56.9)	2.1 (1.49, 2.94)	0.68 (0.47, 1.0)	0.05
Number of ANC visits	1–3 visits	183 (53.2)	161 (42.8)	1.28 (0.82, 1.97)	1.38 (0.84, 2.28)	0.21
	4 and above visits	204 (54.8)	168 (45.2)	1.36 (0.88, 2.10)	1.18 (0.71, 1.96)	0.53
	No	49 (47)	55 (53)	1	1	1
Gravidity	Primigravida	160 (50.3)	158 (49.7)	1	1	1
	Multigravida	276 (55)	226 (45)	0.83 (0.63, 1.1)	2.83 (1.9, 4.21)	≤ 0.01
Gestational age at birth	Below 37 weeks	120 (42.9)	160 (57.1)	1		1
	37–42 weeks	306 (59.5)	208 (40.5)	0.51 (0.38, 0.68)	1.82 (1.31, 2.5)	≤ 0.01
	Above 42 weeks	10 (38.5)	16 (61.5)	1.2 (0.52, 2.74)	0.48 (0.189, 1.22)	0.12
Place of delivery	Home	12 (34.3)	23 (65.7)	1	1	1
	Health institution	424 (54)	361 (46)	0.44 (0.217, 0.91)	2.15 (0.955, 4.82)	0.06
Knowledge of SSC	Poor	252 (63.6)	144 (36.3)	1	1	1
	Good	184 (43.4)	240 (56.6)	2.28 (1.72, 3.02)	2.11 (1.53, 2.92)	≤ 0.01

*ANC, antenatal care; SSC, skin-to-skin contact; CC, cord care*.

#### Determinants of Safe Cord Care Practices

This study pointed out that the age of mothers, educational status, and knowledge of post-partum women on SSC and CC were significantly associated with safe Cord Care (CC) practice at multivariable logistic regression.

In this study age of the mother was significantly associated with safe cord care practice. Mothers aged 20–29 were 2.93 times more likely to practice safe cord care than mothers aged under 19 [AOR = 2.93, 95% CI: 1.24, 6.96]. In this study, mothers with a college education or higher were 1.83 times [AOR = 1.83, 95% CI: 1.08, 3.13] more likely to practice safe cord care than mothers with no formal education. Respondents with good knowledge levels were 11.3 times more likely than respondents with poor knowledge levels to practice safe cord care [AOR = 11.3, 95% CI: (7.49, 17.18)] (Table 4).

**Table 4:** Determinants of cord care practice among post-partum women at public hospitals in eastern Ethiopia, 2021.

**Variable**	**Category**	**Cord care**		**COR 95% CI**	**AOR 95%CI**
		**Clean**	**Unclean**		
Age of mothers	15–19	42 (84)	8 (16)	1	1
	20–29	296 (67.6)	142 (32.4)	2.52 (1.15, 5.50)^*^	2.93 (1.24, 6.96)^*^
	30–39	222 (75.5)	72 (24.5)	1.70 (0.76, 3.79)	2.45 (0.95, 6.31)
	Above 40	28 (73.7)	10 (26.3)	1.87 (0.660, 5.33)	2.65 (0.78, 9.06)
Educational status	No formal education	46 (28.8)	104 (71.2)	1	1
	Primary	32 (24.2)	100 (75.8)	0.66 (0.39, 1.13)	0.90 (0.49, 1.66)
	Secondary	72 (28.6)	180 (71.4)	0.87 (0.56, 1.35)	1.08 (0.65, 1.81)
	Collage and above	82 (28.7)	204 (71.3)	0.87 (0.57, 1.34	1.83 (1.08, 3.13)^*^
Gravidity	Primigravida	94 (29.6)	224 (69.4)	1	1
	Multigravida	138 (27.5)	364 (72.5)	0.9 (0.66, 1.23)	1.18 (0.78, 1.79)
History of newborn loss	No	30 (34.1)	58 (65.9)	1.36 (0.85, 2.17)	1.22 (0.69, 2.14)
	Yes	202 (27.6)	530 (72.4)	1	1
Place of delivery	Home	12 (34.3)	23 (65.7)	1	1
	Health institution	220 (28)	565 (72)	0.74 (0.366, 1.525)	0.65 (0.27, 1.56)
Immediate postnatal counseling	Yes	202 (27.5)	532 (72.5)	0.71 (0.44, 1.14)	1.29 (0.74, 2.25)
	No	30 (34.9)	56 (65.1)	1	1
Knowledge of cord care	Poor	199 (54)	170 (46)	1	1
	Good	52 (11.5)	399 (88.5)	8.98 (6.2, 13.22)^**^	11.3 (7.49, 17.18)^**^

** *P-value ≤ 0.01*.

* *P-value ≤ 0.05*.

## Discussion

Despite numerous benefits of CC and SSC, they are not widely practiced in Ethiopian health facilities; thus, this study aimed to investigate the level of practice and determinants of safe cord care and skin-to-skin contact care among post-partum women in public health facilities.

According to this study, 71.7% practice safe cord care (95% CI: 64.5, 81.7). This is consistent with studies conducted in Sidama, Ethiopia 73%) (28), four Ethiopian regions 65.2%, (29) and Nigeria 77.8% (30). While the findings of this study were higher than that of studies done in Ghana 35.7% (31). In contrast to this, it was lower than previous studies conducted in Nigeria 32.7% (22). This variation could be attributed to differences in sample size and socio-cultural differences among study participants. According to this study, the proportion of skin-to-skin contact care is 53.2% (95% CI: 43.6, 58.8). The findings of this study were consistent with previous studies conducted in Mekelle (43.9%) (32), Sidama, Ethiopia 52% (28), and rural Ethiopia (44.1%) (33). However, this study's findings were higher than those of studies conducted in southern Ethiopia 35.3% (25), four Ethiopian regions 25.8% (29), rural Ghana 10% (34), Gambia 35.7% (14), and Brazil 37.5% (35). The disparity may be due to differences in health facility setup, differences in maternal knowledge, differences in sociodemographic characteristics, and differences in sample size. Our study pointed out that mothers between the ages of 20 and 29 were 2.93 times more likely to practice safe cord care than mothers under the age of 19. The possible justification is that as people get older, their exposure to information grows, which increases the practice of clean cord care. In this study, mothers with a college education or higher were 1.83 times more likely to practice safe cord care than mothers with no formal education. This is in line with the findings of a study conducted in Ghana (31). The possible justification is that as mothers' educational status improves, so will their knowledge of the benefits of clean cord care, which will increase the practice of clean cord care.

When compared to respondents with poor knowledge levels, mothers with good knowledge were 11.3 times more likely to practice safe cord care. This finding is consistent with a study conducted in Nigeria (30). One possible explanation is that mothers who understand the importance of clean cord care (prevention of umbilical cord infection, rapid healing) will practice it. According to a systematic review and meta-analysis, most women applied substances to the cord to prevent infections or help the wound heal, but their practice was not clean, resulting in infection and neonatal death (36). As a result, it is critical to advise post-partum women to practice clean cord care.

This study also revealed that mothers between the ages of 20 and 29, 30–39, and over 40 were 11, 4, and 14 times more likely to practice skin-to-skin contact care than mothers under the age of 19. SSC care practice was 3.70 times more likely in multigravida mothers than in primigravida mothers. The possible justification is that as one's age and childbirth experience increase, so does the likelihood of receiving information about skin-to-skin contact, which in turn plays a pivotal role in enhancing skin-to-skin contact practice. In this study, mothers whose occupations were merchant and self-employed were 0.55 and 0.49 times less likely to practice SSC care, respectively, than mothers whose occupation was a housewife. This is because mothers who work as housewives have more time to access media and quality health care services, and they may be more aware of the benefits of skin-to-skin care practices, which in turn increases the likelihood of skin-to-skin contact practice. Another possible explanation is that because those housewives did not work, they spent most of their time with their newborns, which may have increased the practice of skin-to-skin contact.

The odds of SSC care practice among mothers who had a gestational age of 37–42 weeks at birth were 1.82 times that of mothers who had a gestational age of fewer than 37 weeks. This is due to the increased risk of preterm birth complications such as respiratory distress syndrome, Meconium aspiration syndrome, congenital anomalies, neonatal jaundice, and neonatal sepsis (28, 37–39). As a result, the newborn may be separated from mothers to receive treatments in the neonatal intensive care units, reducing the mother's time to practice skin-to-skin care.

Mothers with good knowledge levels were 3.19 times more likely to practice SSC care as compared to respondents with poor knowledge levels. The finding of the study is consistent with the study done in Gurage, south Ethiopia (16). The reason could be that mothers who have a good understanding of the benefits and drawbacks of skin-to-skin care are more likely to practice it.

### Strength and Limitation of the Study

The strength of the study was the use of a larger sample size, the use of primary data, and the relevance of the topic. The cross-sectional nature of the current study may not indicate a cause-effect relationship, which was one of the study's limitations. The study did not address socio-cultural factors, which can be investigated through qualitative research design.

## Conclusions

The practicing level of safe cord care and skin-to-skin contact was high compared to other studies done in Ethiopia. In this study, the Age of mothers, marital status, occupational status, gestational age at birth, and knowledge of post-partum women on SSC and CC were significantly associated with skin-to-skin contact practice, whereas the age of mothers, educational status, and knowledge of post-partum women on SSC and CC were not significantly associated with SSC. To reduce neonatal mortality due to avoidable umbilical cord infections, clean cord care practice should be strengthened and intensified. Furthermore, skin-to-skin contact practice should be strengthened to enhance the survival of at-risk neonates.

## Data Availability Statement

The original contributions presented in the study are included in the article/supplementary material, further inquiries can be directed to the corresponding author/s.

## Ethics Statement

Ethical approval was taken from the Institutional Health Research Ethics Review Committee (IHRERC) of the College of Health and Medical Sciences of Haramaya University. Written informed consent to participate in this study was provided by the participants for those who are ≥ 18 years old and legal guardian/next kin for those <18 years old.

## Author Contributions

AE and TG conceived the idea and had major roles in the data review, drafting, and writing the result. AD, AA, AB, and YD contributed to data analysis, drafting, and manuscript preparation. All authors read and approved the final version of the manuscript to be published and agreed on all aspects of this work.

## Conflict of Interest

The authors declare that the research was conducted in the absence of any commercial or financial relationships that could be construed as a potential conflict of interest.

## Publisher's Note

All claims expressed in this article are solely those of the authors and do not necessarily represent those of their affiliated organizations, or those of the publisher, the editors and the reviewers. Any product that may be evaluated in this article, or claim that may be made by its manufacturer, is not guaranteed or endorsed by the publisher.

## Abbreviations

ANC: antenatal care
CC: cord care
HFSUH: Hiwot Fana Specialized University Hospital
PP: post-partum
SSC: skin-to-skin contact
WHO: World Health Organization.
